# Filling the gap in central shielding: three-dimensional analysis of the EQD2 dose in radiotherapy for cervical cancer with the central shielding technique

**DOI:** 10.1093/jrr/rrv029

**Published:** 2015-06-10

**Authors:** Tomoaki Tamaki, Tatsuya Ohno, Shin-ei Noda, Shingo Kato, Takashi Nakano

**Affiliations:** 1 Department of Radiation Oncology, Saitama Medical University International Medical Center, 1397-1 Yamane, Hidaka, Saitama 350-1298, Japan; 2 Department of Radiation Oncology, Gunma University Graduate School of Medicine, 3-39-22 Showa-machi, Maebashi, Gunma 371-8511 Japan

**Keywords:** cervical cancer, radiotherapy, brachytherapy, EQD2, central shielding

## Abstract

This study aimed to provide accurate dose distribution profiles of radiotherapy for cervical cancer when treated with the central shielding technique by analysing the composite 3D EQD2 dose distribution of external beam radiotherapy (EBRT) plus intracavitary brachytherapy (ICBT). On a phantom, four patterns of the combinations of whole pelvis irradiation (WP) (4 fields), pelvis irradiation with central shielding technique (CS) [anterior–posterior/posterior–anterior (AP-PA fields), shielding width of 3 or 4 cm] and ICBT using Point-A prescription were created: 30 Gy/15 fractions + 20 Gy/10 fractions + 24 Gy/4 fractions [Plan (30 + 20 + 24)], 40 Gy/20 fractions + 10 Gy/5 fractions + 18 Gy/3 fractions [Plan (40 + 10 + 18)], 40 Gy/20 fractions + 10 Gy/5 fractions + 24 Gy/4 fractions [Plan (40 + 10 + 24)] and 45 Gy/25 fractions + 0 Gy + 28 Gy/4 fractions [Plan (45 + 0 + 28)]. The composite EQD2 dose distributions of the complete treatment were analysed. The Point-A dose of Plan (30 + 20 + 24), Plan (40 + 10 + 18), Plan (40 + 10 + 24) and Plan (45 + 0 + 28) were 78.0 Gy (CS 3 cm)/71.8 Gy (CS 4 cm), 72.1 Gy (CS 3 cm)/69.0 Gy (CS 4 cm), 80.1 Gy (CS 3 cm)/77.0 Gy (CS 4 cm) and 84.1 Gy, whereas it has been previously reported to be 62 Gy, 64 Gy, 72 Gy and 84 Gy, respectively. For all the treatment plans with CS, equivalent or wider coverage of 60 Gy (EQD2) was achieved in the right–left direction, while coverage in the anterior–posterior direction decreased in plans with CS. There were no irregularly ‘cold’ regions around the central target. The use of CS in radiotherapy for cervical cancer resulted in tumor coverage in the lateral direction with doses higher than the previously reported Point-A doses.

## INTRODUCTION

The standard curative radiotherapy for cervical cancer consists of a combination of external beam radiotherapy (EBRT) and intracavitary brachytherapy (ICBT). For EBRT, whole pelvis irradiation (WP) is utilized to cover the primary tumor in the cervix and the lymph node regions in the pelvis. Historically, pelvis irradiation with the central shielding technique (CS) has been used for some of the EBRT to lower the irradiation dose to the bladder and rectum [1–5]. In Japan, CS has long been practised as a standard therapy [6], and its wide use has been reported in previous pattern-of-care studies [7].

The use of CS aims to lower the incidence of toxicities in the bladder and rectum. However, it also reduces the dose delivered to the primary tumor in the cervix and hinders precise assessment of the total dose delivered by the combination of EBRT and ICBT. As a result, the total irradiated dose has often been reported simply by the dose at Point A, with complete omission of the doses delivered by CS [8, 9]. The delivered doses computed in this manner have been shown to be significantly lower than those in other studies that do not use CS [8, 9], and it has been difficult to elucidate the appropriate doses for tumor control despite the superior clinical outcomes with the use of CS. Although it is very important, until recently, 3D dose distribution analysis by the composite dose distribution of EBRT and ICBT has rarely been performed to clarify local control probability and complications after radiation therapy. Nowadays, the use of 3D images in the treatment planning of EBRT and ICBT, and the application of medical imaging software, have enabled dose analysis of different dose fractionations between EBRT and ICBT.

In this study, we used a simple phantom model to analyse the composite dose distribution of radiotherapy (WP, CS and IBCT) for cervical cancer. The composite biologically effective doses were analysed as the 3D dose distributions in equivalent doses delivered in 2-Gy fractions (EQD2). Representative combinations of doses for WP, CS and ICBT in previous reports from Japan and Europe were compared [3, 4, 9, 10].

## MATERIALS AND METHODS

### Simulated radiation therapy plans

Simulated treatment plans for EBRT and brachytherapy (BT) were created on a dosimetric phantom. Previously reported dose schedules were used [3, 4, 9, 10].

The WP fields were 4 orthogonal fields of 18 cm (cranial–caudal) by 18 cm (right–left; RL) by 12 cm (anterior–posterior; AP). The CS fields were the anterior–posterior/posterior–anterior (AP/PA) fields of 18 cm by 18 cm with a central shielding of 3 or 4 cm. The dose prescription point of the WP fields was the center of the box formed by the orthogonal fields, and that of the CS fields was 5 cm left of the center. The physical dose distribution was calculated with the superposition algorithm (Xio ver. 4.50, CMS) using the data for 10-MV beams in our clinic. The CT values of the phantom were replaced with the equivalent value of water. Three patterns of EBRT were created for WP and CS: 30 Gy/15 fractions plus 20 Gy/10 fractions [Plan (30 + 20)]; 40 Gy/20 fractions plus 10 Gy/5 fractions [Plan (40 + 10)]; and 45 Gy/25 fractions plus 0 Gy [Plan (45 + 0)].

BT plans were created using the Fletcher-type tandem and ovoid applicators. The tandem length was 6 cm, and the distance between the ovoids was 3 cm. The standard loading pattern of our institution, which results in a pear-shaped isodose distribution, was used. Three plans were created for this study: 18 Gy/3 fractions (BTPlan 18/3), 24 Gy/4 fractions (BTPlan 24/4) and 28 Gy/4 fractions (BTPlan 28/4). The doses were prescribed to Point A.

For total treatment, the following four combinations were created: Plan (30 + 20) plus BTPlan 24/4 [Plan (30 + 20 + 24)], Plan (40 + 10) plus BTPlan 18/3 [Plan (40 + 10 + 18)], Plan (40 + 10) plus BTPlan 24/4 [Plan (40 + 10 + 24)], and Plan (45 + 0) plus BTPlan (28/4) [Plan (45 + 0 + 28)] (Table 1). The Point-A dose in each plan was previously reported to be 62 Gy, 64 Gy, 72 Gy and 84 Gy, respectively.

**Table 1.** Summary of combinations of whole pelvis irradiation (WP), pelvis irradiation with central shielding technique (CS) and brachytherapy (BT) analysed in this study

	Plan (30 + 20 + 24) [4, 9]	Plan (40 + 10 + 18) [9]	Plan (40 + 10 + 24) [4]	Plan (45 + 0 + 28) [3, 10^a^]
WP	30 Gy/15 fr	40 Gy/20 fr	40 Gy/20 fr	45 Gy/25 fr
CS	20 Gy/10 fr	10 Gy/5 fr	10 Gy/5 fr	0 Gy
BT	24 Gy/4 fr	18 Gy/3 fr	24 Gy/4 fr	28 Gy/4 fr
Previously reported total Point-A dose (EQD2)	62 Gy	64 Gy	72 Gy	∼84 Gy

^a^HR-CTV D90 prescription.

### Evaluation of composite dose distribution

The plans of EBRT and BT were evaluated using treatment analysis software (MIM Maestro, ver. 6.2, MIM Software). Physical doses were converted to doses in EQD2 using an alpha/beta ratio of 10 Gy, and the composite dose distributions of EBRT plans, BT plans and EBRT plans plus BT plans (total doses) were evaluated with the spatial dose distributions in EQD2.

## RESULTS

**Fig. 1.**
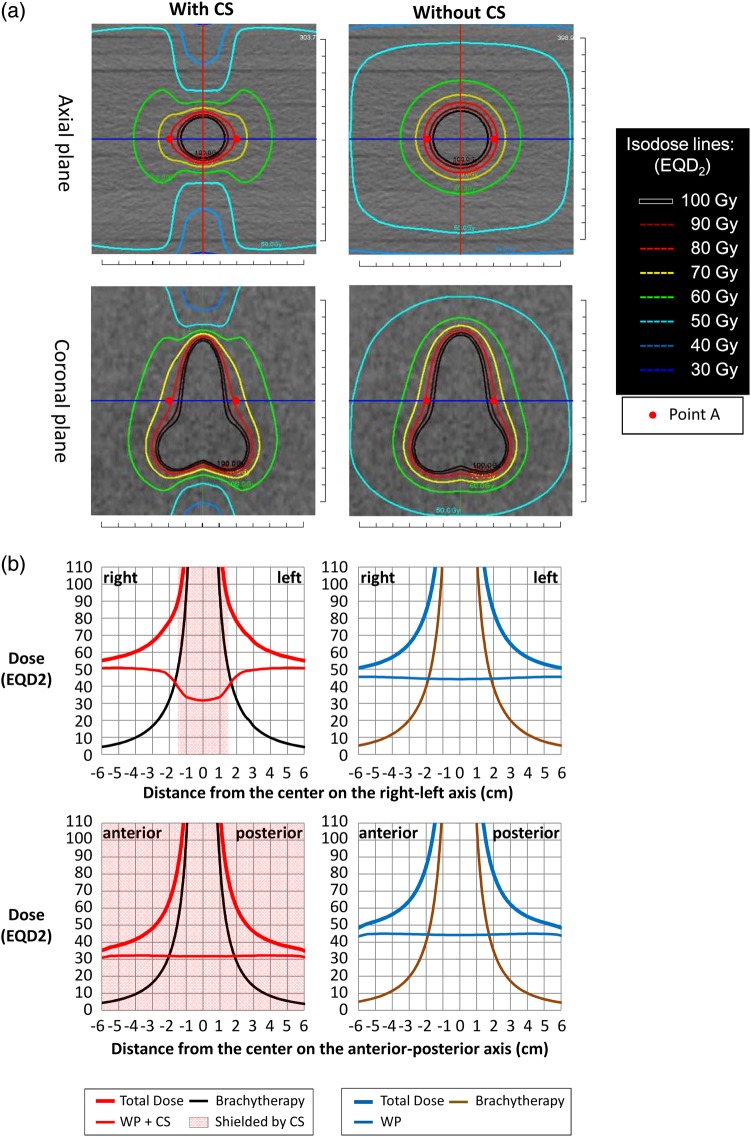
(**a**) The composite EQD2 dose distributions of WP 30 Gy/15 fractions + CS 20 Gy/10 fractions + BT 24 Gy/4 fractions (CS 3 cm) and WP 45 Gy/25 fractions + BT 28 Gy/4 fractions. The composite EQD2 distributions on the axial and coronal images at the level of Point A are shown. (**b**) The doses of EBRT and ICBT and the total dose in EQD2 on the RL axis [blue lines in (a)] and on the AP axis [red lines in (a)] are shown.

**Fig. 2.**
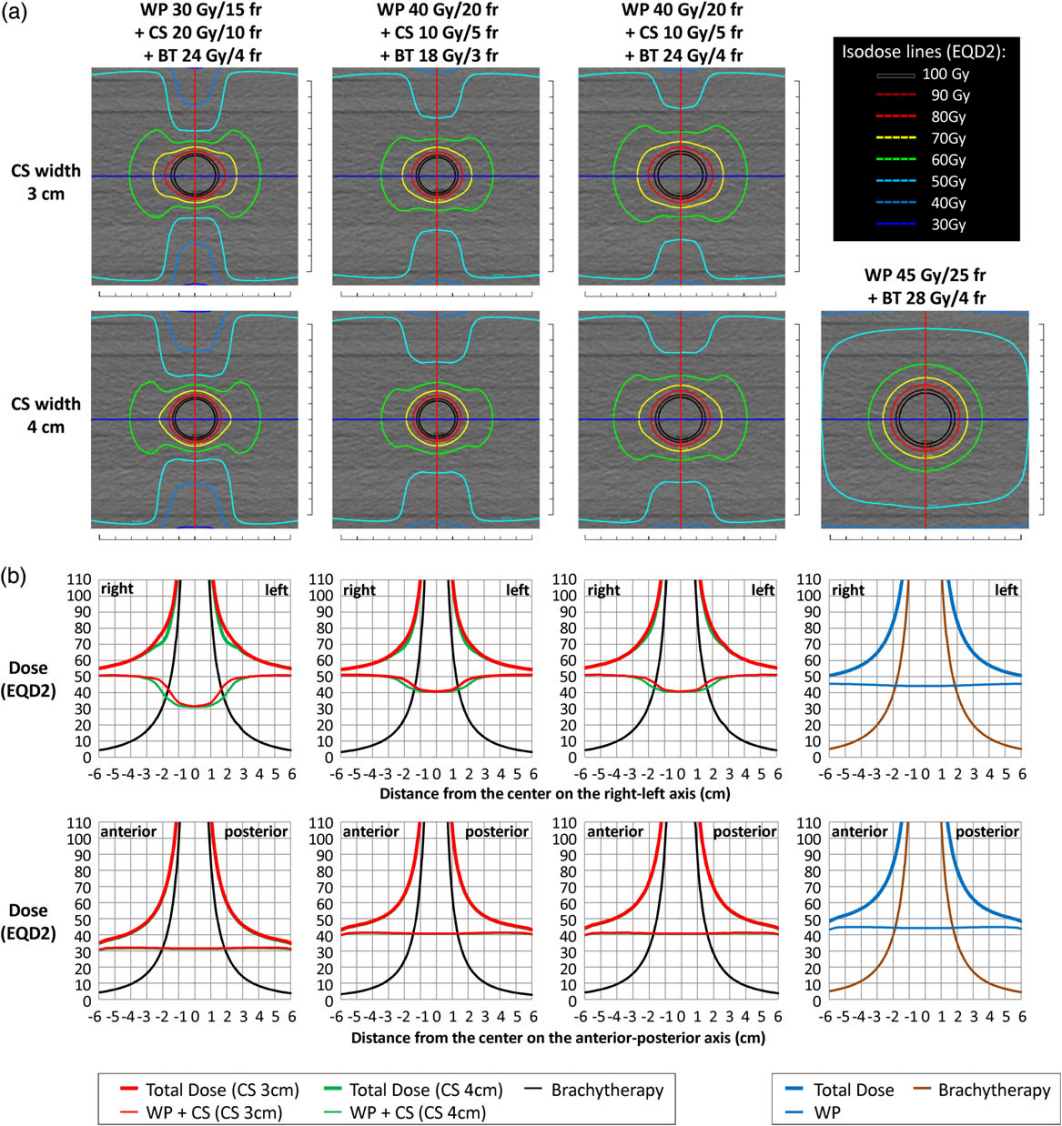
(**a**) The composite EQD2 dose distributions of WP 30 Gy/15 fractions + CS 20 Gy/10 fractions + BT 24 Gy/4 fractions (CS 3 or 4 cm) (far left column), WP 40 Gy/20 fractions + CS 10 Gy/5 fractions + BT 18 Gy/3 fractions (CS 3 or 4 cm) (near left column), WP 40 Gy/20 fractions + CS 10 Gy/5 fractions + BT 24 Gy/4 fractions (CS 3 or 4 cm) (near right column) and WP 45 Gy/25 fractions + BT 28 Gy/4 fractions (far right column). The composite EQD2 distributions on the axial images at the level of Point A are shown. (**b**) The doses of EBRT and ICBT and the total dose in EQD2 on the RL axis [blue lines in (a)] and on the AP axis [red lines in (a)] are shown. The EBRT doses and the total doses of CS 4 cm (green lines) are mostly overlaid by those of CS 3 cm (red lines), except around the borders of the central shielding.

The axial and coronal planes of the composite EQD2 dose distributions for Plan (30 + 20 + 24) (CS 3 cm) and Plan (45 + 0 + 28) are shown in Fig. 1a. All the other EQD2 distributions are shown in Fig. 2a. In the plans with CS, the 50-Gy isodose line extended laterally and covered the volumes that would include parametrial tissues and pelvic lymph nodes. In the central regions that were shielded by the CS fields, the doses decreased to ∼30 Gy or 40 Gy near the anterior and posterior edges of the volumes irradiated by the WP fields.

The areas covered with 60 Gy (green isodose lines) extended laterally in the regions irradiated by the CS fields. In particular, in Plan (30 + 20 + 24), the 60-Gy isodose line formed a butterfly-like shape on the axial plane (Fig. 1a). Figure 2a reveals that the lateral extensions of the 60-Gy isodose lines were larger in Plan (30 + 20 + 24) and Plan (40 + 10 + 24) than in Plan (45 + 0 + 28). The lateral extension of the 60-Gy isodose line in Plan (40 + 10 + 18) was similar to that of Plan (45 + 0 + 28). On the other hand, the AP extensions of the 60-Gy isodose lines resulted in a diameter >6 cm in Plan (45 + 0 + 28) and became smaller in order of Plan (40 + 10 + 24), Plan (40 + 10 + 18) and Plan (30 + 20 + 24).

Figure 1b shows the graphs of the EQD2 doses of the EBRT, BT and total plans on the RL axes (blue lines in Fig. 1a) and AP axes (red lines in Fig. 1a) on the axial plane at the level of Point A. The graphs for all the plans are shown in Fig. 2b. In the RL axes, the dose distributions of the EBRT plan of Plan (30 + 20) showed a gradient of ∼50–30 Gy within 1–2.5 cm of the center (Fig. 1b, the upper row). On the other hand, the dose distribution of BTPlan 24/4 showed a steeply rising gradient of ∼20–90 Gy within 1–2.5 cm of the center. Thus, the gap of the EBRT doses that resulted from the use of CS was compensated for by the BT doses, and the composite dose distributions of EBRT plus BT resulted in increasing dose distributions of 70–120 Gy within 1–2.5 cm of the center. When CS widths of 3 and 4 cm were compared, the dose differences were most prominent (∼10 Gy) at ∼2 cm from the center (Fig. 2b). In the AP axes, the dose distributions of WP with CS were almost uniformly equal to the WP dose of 30 or 40 Gy, with small contributions from the CS irradiation; thus, the composite dose distributions were a simple addition of the uniform EBRT doses and the BT doses (Fig. 1b, lower row).

The doses at Point A and the lengths covered by 50 Gy to 100 Gy (in increments of 10 Gy) in the RL and AP axes on the axial plane at the level of Point A are shown in Table 2. The doses at Point A for Plan (30 + 20 + 24), Plan (40 + 10 + 18), Plan (40 + 10 + 24) and Plan (45 + 0 + 28) were 78.0 Gy (CS 3 cm)/71.8 Gy (CS 4 cm), 72.1 Gy (CS 3 cm)/69.0 Gy (CS 4 cm), 80.1 Gy (CS 3 cm)/77.0 Gy (CS 4 cm) and 84.1 Gy, respectively. The width (RL direction) covered by 70 Gy was 5.2–5.3 cm for Plan (30 + 20 + 24) (CS 3 cm) and Plan (40 + 10 + 24), and this was similar to that for Plan (45 + 0 + 28). The widths covered by 70 Gy in Plan (30 + 20 + 24) (CS 4 cm) and Plan (40 + 10 + 18) were smaller. The widths covered by doses <70 Gy were wider in Plan (30 + 20 + 24) and Plan (40 + 10 + 24) than in Plan (45 + 0 + 28). The widths covered by doses <70 Gy were similar in Plan (40 + 10 + 18) and Plan (45 + 0 + 28). On the other hand, the widths covered by doses >70 Gy (80–100 Gy) were wider in Plan (45 + 0 + 28) than in other plans (Table 2).

**Table 2.** The doses at Point A, the lengths on the anterior–posterior (AP) and right–left (RL) axes covered by 50–100 Gy (EQD2) on the axial plane of Point A, and the volume covered by 50–100 Gy (EQD2)

Plan	Plan (30 + 20 + 24)				Plan (40 + 10 + 18)				Plan (40 + 10 + 24)				Plan (45 + 0 + 28)	
Center shielding width	3 cm		4 cm		3 cm		4 cm		3 cm		4 cm			
Doses at Point A	78.0 Gy		71.8 Gy		72.1 Gy		69.0 Gy		80.1 Gy		77.0 Gy		84.1 Gy	
Axis	AP	RL	AP	RL	AP	RL	AP	RL	AP	RL	AP	RL	AP	RL
Lengths covered by 100 Gy (cm)	2.4	2.6	2.4	2.5	2.2	2.3	2.2	2.2	2.7	2.8	2.7	2.7	3.2	3.2
Lengths covered by 90 Gy (cm)	2.7	3.0	2.7	2.8	2.5	2.6	2.5	2.5	3.0	3.3	3.0	3.1	3.6	3.7
Lengths covered by 80 Gy (cm)	3.0	3.8	3.0	3.2	2.9	3.2	2.9	3.0	3.4	4.0	3.4	3.7	4.1	4.3
Lengths covered by 70 Gy (cm)	3.5	5.2	3.5	4.5	3.4	4.3	3.4	3.9	4.1	5.3	4.1	5.2	5.0	5.3
Lengths covered by 60 Gy (cm)	4.2	8.2	4.2	8.2	4.5	7.0	4.4	6.9	5.3	8.3	5.3	8.3	6.7	7.1
Lengths covered by 50 Gy (cm)	5.4	>12	5.4	>12	7.0	>12	6.9	>12	8.2	>12	8.1	>12	11.2	>12
Volume covered by 100 Gy (cm^3^)	47.7		45.5		38.9		38.0		54.2		52.8		70.9	
Volume covered by 90 Gy (cm^3^)	59.0		55.4		48.8		47.3		68.3		65.9		88.2	
Volume covered by 80 Gy (cm^3^)	77.9		71.0		65.2		62.3		92.3		87.5		116.2	
Volume covered by 70 Gy (cm^3^)	118.1		101.7		99.1		91.5		143.2		132.1		169.1	
Volume covered by 60 Gy (cm^3^)	266.3		221.3		218.9		192.4		329.1		296.5		305.5	
Volume covered by 50 Gy (cm^3^)	2389.4		2228.9		2464.5		2333.8		2609.2		2489.1		1257.7	

The thickness (AP direction) covered by each dose (50–100 Gy) increased from Plan (40 + 10 + 18)/Plan (30 + 20 + 24) to Plan (40 + 10 + 24) to Plan (45 + 0 + 28). For example, the thicknesses covered by 70 Gy in Plan (40 + 10 + 18), Plan (30 + 20 + 24), Plan (40 + 10 + 24) and Plan (45 + 0 + 28) were 3.4 cm, 3.5 cm, 4.1 cm and 5.0 cm, respectively. In addition, the differences became larger in the lower dose ranges (Table 2). Table 2 also shows volumes covered with 50–100 Gy by all of the plans. Compared with Plan (45 + 0 + 28), the irradiated volumes in the range of 100–70 Gy were smaller in Plan (30 + 20 + 24), Plan (40 + 10 + 18) and Plan (40 + 10 + 24). In contrast, in the range of 60–50 Gy, the size of the irradiated volume remained similar or increased in Plan (30 + 20 + 24), Plan (40 + 10 + 18) and Plan (40 + 10 + 24) (Table 2).

## DISCUSSION

While radiotherapy for cervical cancer with the use of CS has been implemented clinically for decades, analysis of the composite dose distribution of EBRT and ICBT has not been reported fully [5]. Nakano *et al*. reported that the 10-year cause-specific survival rates for cervical cancer patients treated with radiotherapy alone were 89%, 74% and 59% for Stage IB, II and III, respectively, whereas the 10-year actuarial rates of major complications were 4.4% in the rectosigmoid colon, 0.9% in the bladder and 3.3% in the small intestines [4]. Thus, the treatment of cervical cancer with the use of CS has yielded comparable or superior clinical outcomes with low incidence of major complications, compared with other reports without the use of CS [4].

The purpose of the use of CS in Japan has been to lower the dose to the rectum and bladder and to provide the same dose to the parametrial tissue as without CS. Its possible disadvantage is that the dose to the cervix could also be reduced. Our analysis revealed that the area covered by >70 Gy (EQD2) was larger when Plan (45 + 0 + 28) was applied. On the other hand, the width (RL direction) covered by <70 Gy (EQD2) was larger when the plans with CS were applied with BT Plan (24/4). In addition, the plans with CS did not result in ‘cold’ regions in the RL axis (Fig. 2), and the EQD2 coverage of the target is consequently smaller in the AP axis when CS is applied (Table 2). The geometric characteristics of the dose distributions are also reflected in the difference in the values of the total volumes irradiated with 50–100 Gy (Table 2). Our results indicate that, while the treatment plans using CS may be able to provide comparable tumor coverage in the RL direction, when treating large tumors, dose optimization may be necessary in order to provide an adequately high dose to the tumor volume in the AP direction.

In this study, the composite doses delivered at Point A are shown in Table 2. These values indicate that omission of the CS doses substantially underestimates the actual doses delivered to the central tumor by these treatment plans, at least in the RL direction. In previous clinical studies, the Point-A dose for Plan (30 + 20 + 24) was reported to be 62 Gy (EQD2) [9], whereas the present study showed that the actual doses would be 78.0 Gy (CS 3 cm) or 71.8 Gy (CS 4 cm). When dose–effect relationships are discussed in comparison with other studies [11], it should be considered that the tumor coverage in the lateral direction (at Point A) may be as high as these values. The 3D composite dose distribution analysis for CS with ICBT clearly demonstrated that the primary tumor at the cervix was provided with substantial doses from ICBT, and that the doses to the parametrical region were not reduced significantly by the use of CS.

The discrepancy between the Point-A doses of the present study and those of previous studies is due to the dose delivered by CS. Tharavichitkul *et al.* studied the effect of CS of 6 Gy/3 fractions and a parametrial boost of 6 Gy/3 fractions after WP of 44 Gy/22 fractions on the Point-A doses and reported that the dose contribution was ∼10 Gy (CS 3 cm) and 6 Gy (CS 4 cm) in EQD2 [12]. Although the treatment schedule utilized was different from that of this study, the ratio of the dose contribution from CS was similar. As seen in Fig. 1 and Fig. 2, the doses delivered with CS compensated for the decrease in the doses delivered by BT in the lateral direction, while the increase in the total doses in the AP direction remained minimal.

The limitation of this phantom study is that the variability in positions of target volumes, organs at risk (OARs) and applicators was not considered. Our provisional analysis showed that a ‘cold’ spot may start to appear when the shift of 0.5 cm in the BT position occurs with a CS width of 4 cm and when the shift of 1.0 cm occurs with a CS width of 3 cm (Supplementary Fig. 1), but other factors such as applicator angle and interfractional variability may also come into consideration. Fenkell *et al.* reported that the use of ‘parametrial boost’ would result in an unpredictable dose, particularly to the OARs, because of the positional variability of OARs and applicators [13]. Because the total doses given in this study and in the actual clinical practice in Japan are lower in comparison with the study by Fenkell *et al*., the sparing effect of bladder and rectum appear to be more evident in this analysis. However, the effect of the positional variability of OARs and applicators and analysis of the dose with respect to normal tissue toxicities based on an alpha/beta ratio of 3 Gy are warranted. In addition, the use of image-guided BT has led to reporting doses in volumes using the GEC-ESTRO recommendation [14], the practice of prescribing BT doses by these parameters [10], and excellent clinical results [15]. This study utilized the Point-A dose for prescription and analysis in order to provide a reference for analysis of previously reported clinical studies. However, our observation of the comprehensive EQD2 dose distribution indicated that the composite dose distributions were complex and should not be described simply with single-point doses, but rather should be analysed as spatial dose distributions or with volumetric parameters in dose–volume histograms. Nevertheless, it is reasonable that this analysis can provide valuable and basic information for analysis of the dose distributions of previous reports and the discussion of future therapeutic approaches for cervical cancer.

In conclusion, 3D composite dose distribution analysis plays a significant role in the correct understanding of the dose distributions of the combination of EBRT and ICBT for cervical cancer. The use of CS in radiotherapy for cervical cancer resulted in tumor coverage in the lateral direction with doses higher than the previously reported Point-A doses, with no irregularly ‘cold’ regions around the central target. While the bladder and rectum can be spared from excessive irradiation, the dose coverage of the tumor in the AP direction becomes smaller than that in the RL direction as a result.

## SUPPLEMENTARY DATA

Supplementary data are available at the *Journal of Radiation Research* online.

## FUNDING

This work was supported by MEXT KAKENHI Grant Number 25861070. Funding to pay the Open Access publication charges for this article was provided by MEXT KAKENHI Grant Number 25861070.

## Supplementary Material

Supplementary Data
