# Supplementary material for: Filling the gap in central shielding: three-dimensional analysis of the EQD2 dose in radiotherapy for cervical cancer with the central shielding technique
Source: J Radiat Res. 2015 Jun 10;56(5):804–10. doi: 10.1093/jrr/rrv029 (PMC4576998; doi:10.1093/jrr/rrv029)
Supplement: Supplementary Data [file supp_56_5_804__index.html]

Filling the gap in central shielding: three-dimensional analysis of the EQD2 dose in radiotherapy for cervical cancer with the central shielding technique — Supplementary Data 

# Filling the gap in central shielding: three-dimensional analysis of the EQD2 dose in radiotherapy for cervical cancer with the central shielding technique

## Supplementary Data

Supplementary Data

- Supplementary Data - Docx file
- Supplementary Figure 1 - tif file
