# Supplementary material for: Filling the gap in central shielding: three-dimensional analysis of the EQD2 dose in radiotherapy for cervical cancer with the central shielding technique
Source: J Radiat Res. 2015 Jun 10;56(5):804–10. doi: 10.1093/jrr/rrv029 (PMC4576998; doi:10.1093/jrr/rrv029)
Supplement: Supplementary Data [file supp_rrv029_rrv029supp.docx]

Supplementary Fig. 1 The doses of EBRT and ICBT and the total dose in EQD2 on the RL axis with shift of ICBT position: the composite EQD2 dose distributions of (upper row) WP 30 Gy/15 fractions + CS 20 Gy/10 fractions + BT 24 Gy/4 fractions (CS 3 or 4 cm), (middle row) WP 40 Gy/20 fractions + CS 10 Gy/5 fractions + BT 18 Gy/3 fractions (CS 3 or 4 cm) and (lower row) WP 40 Gy/20 fractions + CS 10 Gy/5 fractions + BT 24 Gy/4 fractions (CS 3 or 4 cm). The shift of 0.5 cm (left column) and 1.0 cm (right column) are shown.
