# Supplementary figures and images for: Filling the gap in central shielding: three-dimensional analysis of the EQD2 dose in radiotherapy for cervical cancer with the central shielding technique
Source: J Radiat Res. 2015 Jun 10;56(5):804–10. doi: 10.1093/jrr/rrv029 (PMC4576998; doi:10.1093/jrr/rrv029)

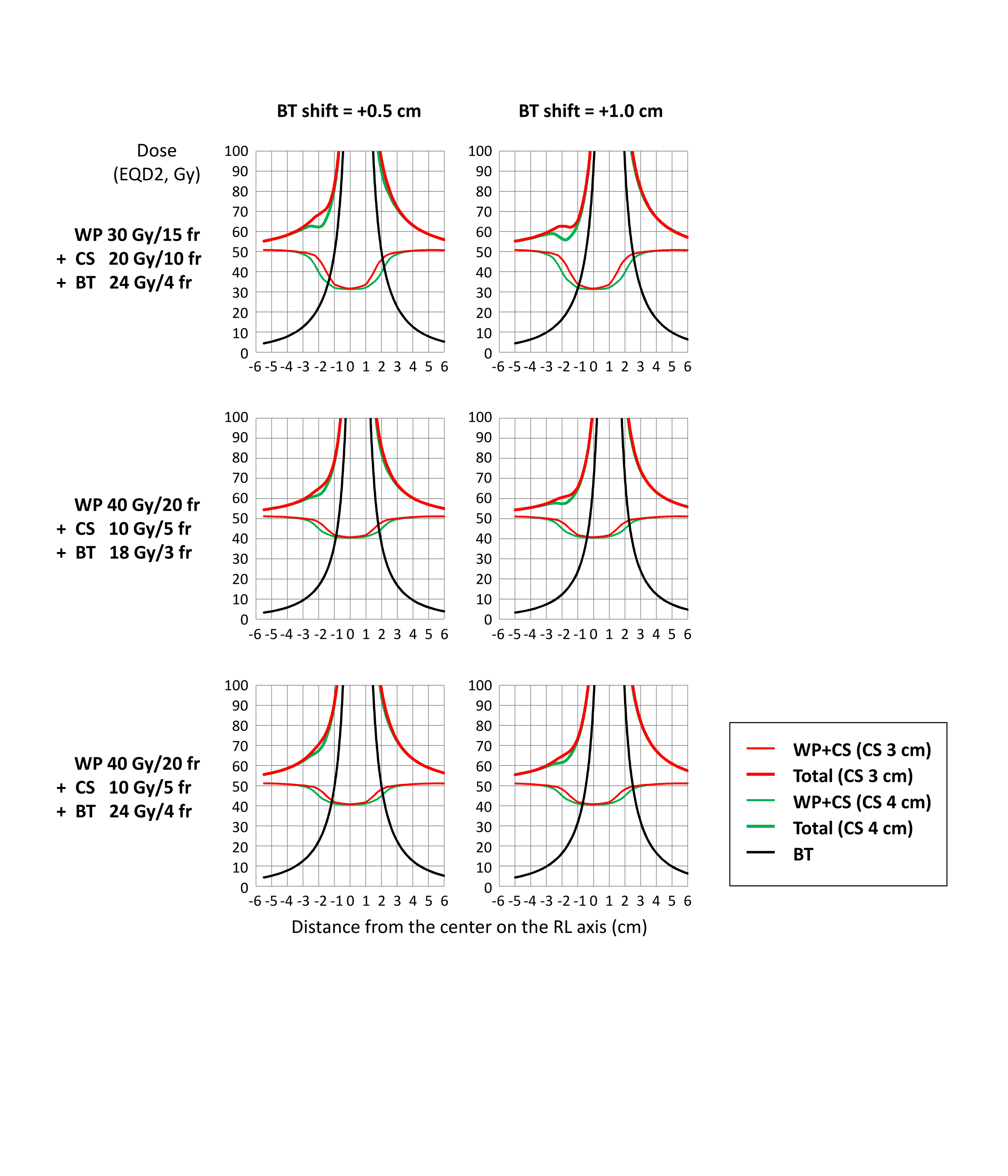

Supplement: Supplementary Data [file supp_rrv029_rrv029supp_fig1.tif]
